# Supplementary material for: Axonal chemokine-like Orion induces astrocyte infiltration and engulfment during mushroom body neuronal remodeling
Source: Nat Commun. 2021 Mar 23;12:1849. doi: 10.1038/s41467-021-22054-x (PMC7988174; doi:10.1038/s41467-021-22054-x)
Supplement: Supplementary file 1 — Supplementary Information [file 41467_2021_22054_MOESM1_ESM.pdf]

Supplementary Materials for

Axonal chemokine-like Orion induces astrocyte infiltration and engulfment during mushroom body neuronal remodeling

Ana Boulanger[1*], Camille Thinat[1], Stephan Züchner[2], Lee G. Fradkin[3], Hugues Lortat-Jacob[4] and Jean-Maurice Dura[1*]

*e-mail:ana.boulanger@igh.cnrs.fr; jean-maurice.dura@igh.cnrs.fr

Supplementary Fig. 1 to 10
Supplementary list of fly strains
Supplementary Table 1

Supplementary Fig.1

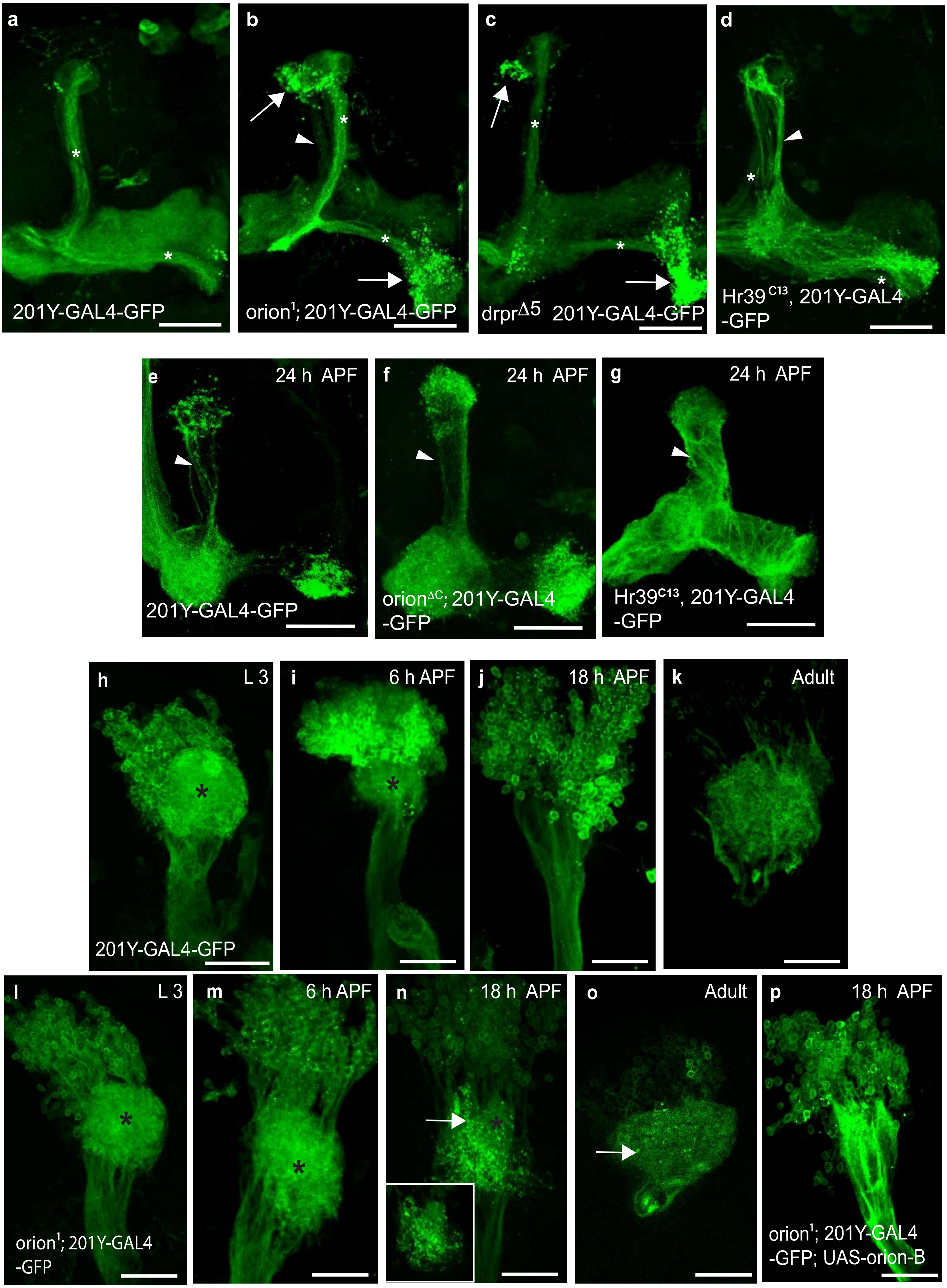

Supplementary Fig. 1. The *orion* gene is necessary for γ axon and dendritic remodeling. **a-d**, Expression of *201Y-GAL4*-driven *UAS-mCD8-GFP* (green) in adult MB γ axons. In adults, this GAL4 line also labels the αβ-core axons indicated here by asterisks. Wild-type (**a**), $orion^{1}$ (**b**), $draper^{\Delta 5}$ (**c**) and $Hr39^{C13}$ (**d**) (n = 22, 26, 30 and 4 MBs). The glial phagocytic receptor Drpr is required for MB remodeling [13]. Note the significant similarity between the $orion^{1}$ and $draper^{\Delta 5}$ phenotypes with respect to the distribution and the amount of axonal debris remaining (arrows in **b** and **c**); they differ by the presence of unpruned γ axons only in $orion^{1}$ (arrowhead in **b**; see also Fig.1b). In addition, the $draper^{\Delta 5}$ phenotype is observed only in very young flies [13]. In contrast, the $orion^{1}$ phenotype persists throughout adult life at least up to one month old in $orion^{1}$ males. Expression of *Hr39* in γ neurons results in only unpruned γ axons (arrowhead) without debris (**d**). In this case, the pruning process is completely blocked due to the *EcR-B1* down-regulation by Hr39 thus precluding the generation of axon debris[43]. **e-g**, Expression of *201Y-GAL4*-driven *UAS-mCD8-GFP* (green) in γ neuron axons at 24 h APF. γ axon development was observed in wild-type (**e**), $orion^{\Delta C}$ (**f**) and $Hr39^{C13}$ (**g**) as indicated. In wild-type (**e**), only some scattered γ axons are still unpruned (arrowhead). Additional unpruned fascicles of axons (arrowhead) are apparent in $orion^{\Delta C}$ (compare **f** with **e**). Note the massive presence of unpruned γ axons (arrowhead) in $Hr39^{C13}$ (**g**) , where the γ axon-intrinsic fragmentation process is blocked. However, since the axon-intrinsic fragmentation process is still functional in $orion^{\Delta C}$, the number of these unpruned axons is much lower than in $Hr39^{C13}$ (n =10 MBs for each developmental stage). **h-p**, Expression of *201Y-GAL4*-driven *UAS-mCD8-GFP* (green) in γ neuron dendrites (black asterisks) during development. Wild-type control (**h-k**) and $orion^{1}$ (**l-p**) γ dendrites are compared at L3, 6 h APF, 18 h APF and adult as indicated. Note the presence of intact larval γ dendrites in $orion^{1}$ (asterisk in **n** compared to wild-type (**j**) at 18 h APF and the persistence of dendrite debris in $orion^{1}$ at 18 h APF (arrow in **n** as well as in adult (arrow in **o**). A confocal plane of a dendrite region containing larval dendrite debris (brilliant dots) is enclosed by a rectangle in **n**. **p**, The $orion^{1}$ unpruned dendritic phenotype is rescued by expression of *UAS-orion-B* at 18 h APF. Because MB calyx is already infiltrated by processes of larval astrocytes, it is possible that, for dendrite pruning, Orion is only required for glial engulfment and not for glial infiltration. All the pictures are confocal Z-projections (n = 8 MBs for each developmental stage). Scale bars are 40 μm. Genotypes are listed in Supplementary list of fly strains.

Supplementary Fig. 2

Presence of unpruned axons in ≥ one-week-old adults

a

	MB	None	Weak	Strong
WT	25	25	0	0
Hr39	22	0	0	22
orionΔC	22	0	22	0
orion1	20	0	20	0
orionRNAi	34	0	34	0
drprΔ5	22	20	2	0

None **Weak** **Strong**

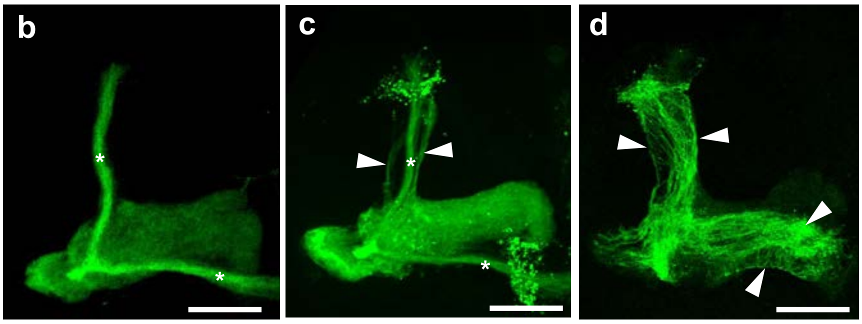

Presence of axon debris in ≥ one-week-old adults

e

	MB	None	Mild	Intermediary	Strong
WT	25	25	0	0	0
Hr39	22	22	0	0	0
orionΔC	22	0	0	0	22
orion1	20	0	0	0	20
orionRNAi	34	34	0	0	0
drprΔ5	22	16	2	2	2

None **Mild** **Intermediary** **Strong**

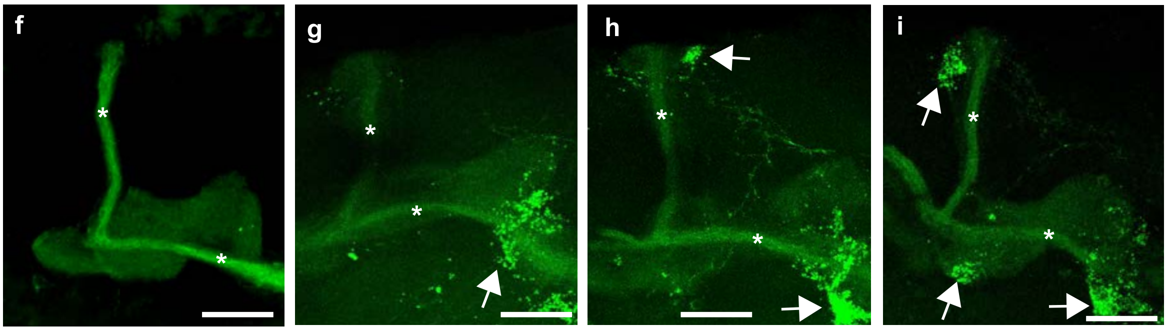

Presence of axon debris in ≤ 2 h-old adults

j

	MB	Scattered dots	Mild	Intermediary	Strong
WT	10	10	0	0	0
orionΔC	12	0	0	0	12
drprΔ5	73	40	11	4	18

None (scattered dots) **Mild** **Intermediary** **Strong**

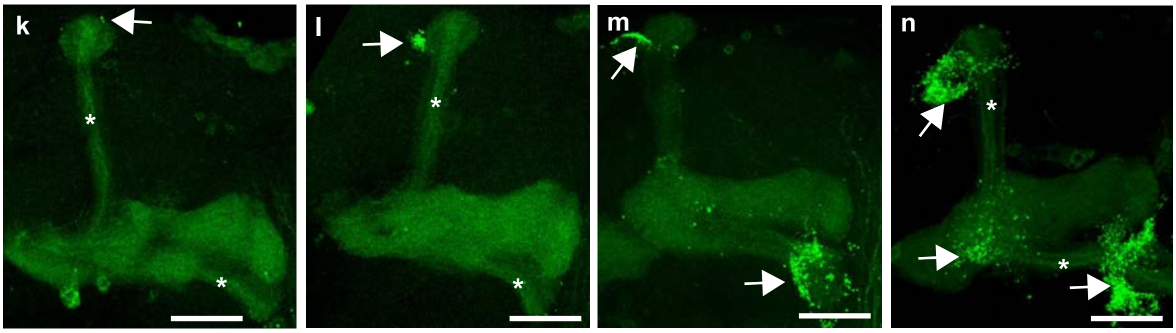

Supplementary Fig. 2. Unpruned axons and axon debris phenotypes. Tables **a**, **e** and **j** show quantitation of the unpruned axon (**a**) and axon debris (**e, j**) and are described in Table I. γ neurons are visualized by the expression of *201Y-GAL4* driven *UAS-mCD8-GFP* (green). In adults, this GAL4 line also labels the αβ-core axons indicated here by asterisks. Unpruned axons are labeled by arrowheads in **b** ("None") in **c** ("Weak") and in **d** ("Strong"). Axon debris are ranked as "None" (**f**), "None (scattered dots)" (**k**), "Mild" (**g, l**), "Intermediate" (**h, m**) and "Strong" (**i, n**) and are labelled by arrows in **g-n**. These dots likely correspond to yet uncleared axon debris **(j, k**). Scale bars are 30 μm. Genotypes are listed in Supplementary list of fly strains.

Supplementary Fig.3

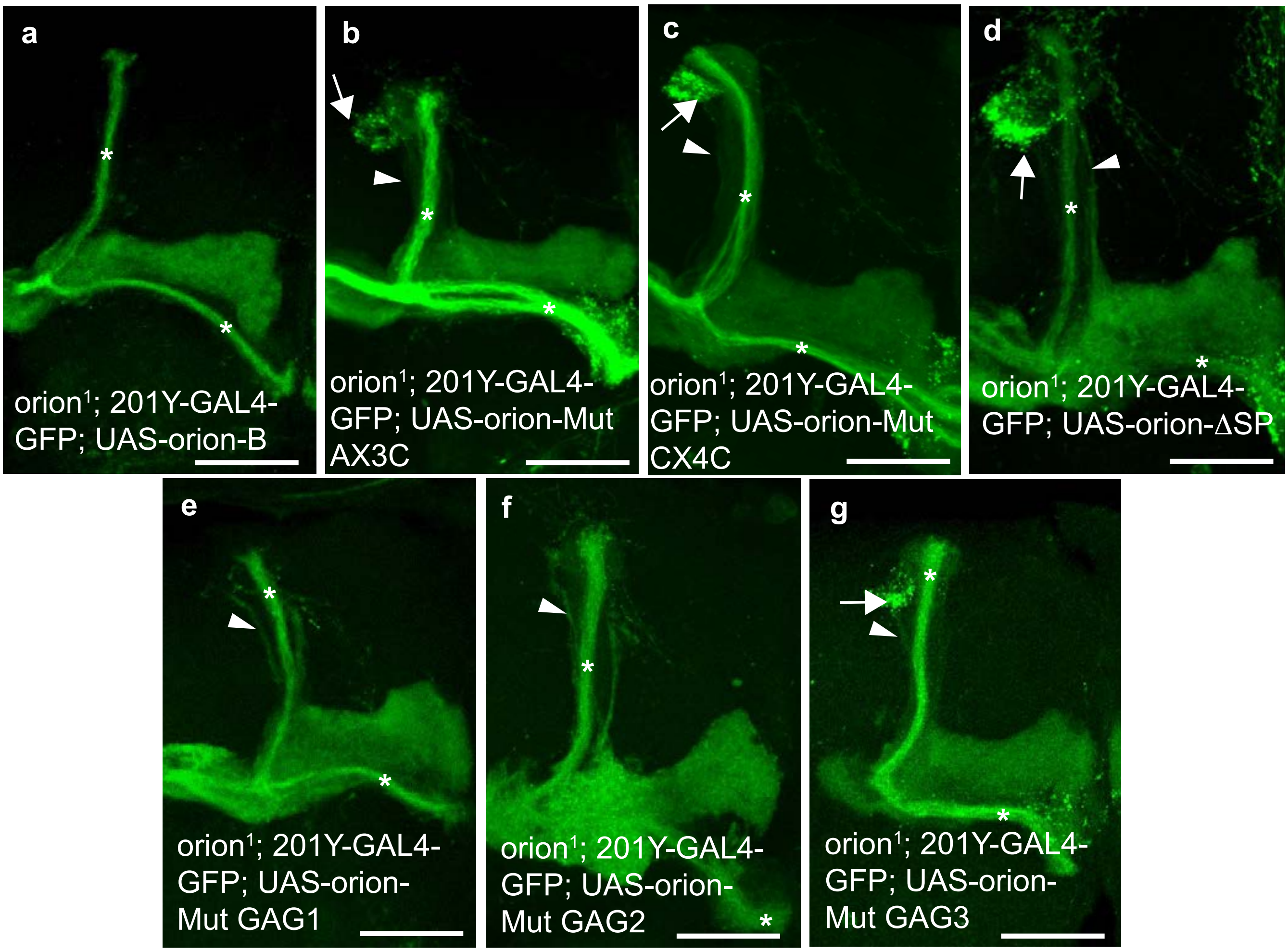

h

	MB	UP	Debris	WT
control	100	0	0	100
orion1	100	100	100	0
orion1 + orion-B WT	387	0	0	387
orion1 + ΔSP	27	27	27	0
orion1 + AX3C	20	20	20	0
orion1 + CX4C	20	20	20	0
orion1 + GAG1	106	46	0	60
orion1 + GAG2	122	52	0	70
orion1 + GAG3	118	112	112	6
orion-RNAi	34	34	0	0
orion-RNAi + EcRB1	26	2	0	24
orion-RNAi + control	20	20	0	0

i

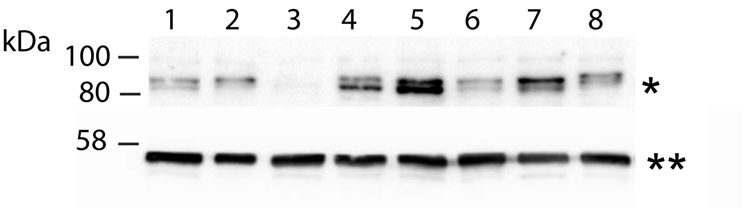

j

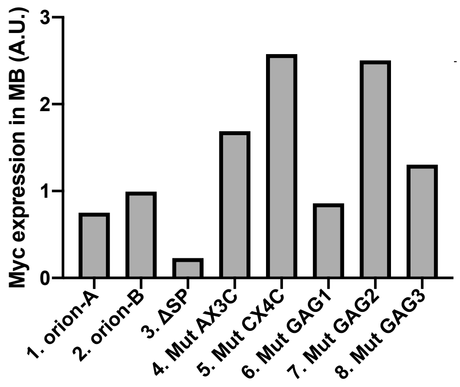

Supplementary Fig. 3. The CX_3C motif, the GAGs sites and the SP domain are required for the Orion pruning function. **a-g**, The expression of *201Y-GAL4* driven *UAS-mCD8-GFP* (green) is shown in adult MBs in which expression of wild-type *UAS-orion-B* (**a**) (n = 387 MBs) or *UAS-orion-B* containing different mutations (Mut, **b-g**) was induced in *orion[1]*. *UAS-orion-B* contains each of the following mutations: at the CX_3C site (AX_3C in **b**, n = 20 MBs; CX_4C in **c**, n = 20 MBs), absence of signal peptide (ΔSP in **d**, n = 27 MBs), at the GAG1 site (EKRTERTLKILKD into EAATEATLAILAD in **e**, n = 106 MBs), at the GAG2 site (VKRNRV into VAANAV in **f**, n = 122 MBs), at the GAG3 site (ARREKLRL into AAAEALAL in **g**, n = 118 MBs). Unpruned γ axons are labelled by arrowheads, uncleared debris are labelled by arrows and αβ core axons are labeled by asterisks. Note that debris are absent in **e** and **f**. Scale bars are 40 μm. **h**, Quantitation of the phenotypes are shown. MB: total number of MBs analyzed; UP: number of MBs containing unpruned γ axons; Debris: number of MBs containing uncleared debris; WT: number of wild-type looking MBs. Genotypes are listed in Supplementary list of fly strains. **i**, Western blot, incubated with an anti-Myc antibody, displaying the Orion-Myc expression levels (single asterisk) produced by the different *UAS-orion-myc* constructs driven by *201Y-GAL4* and the tubulin levels in each genotype (double asterisk) as a control. Proteins were extracted from L3 brains. Lane 1: *orion-A*; lane 2: *orion-B*; lane 3: *orion-B-ΔSP*; lane 4: *orion-B-Mut AX3C*; line 5: *orion-B-Mut CX4C*; line 6: *orion-B-Mut GAG1*; lane 7: *orion-B-Mut GAG2*; lane 8: *orion-B-Mut GAG3*. Source data are provided as a Source Data file. **j**, Orion-Myc band expression levels are shown in arbitrary units (A.U.) which are calculated as a ratio of the Myc level to the loading control tubulin level for each genotype. Note that all of the proteins are expressed at similar or higher levels relative to Orion-B except Orion-ΔSP whose expression is lower likely due to protein destabilization resulting from the lack of the signal peptide. Source data are provided as a Source Data file.

Supplementary Fig.4

A

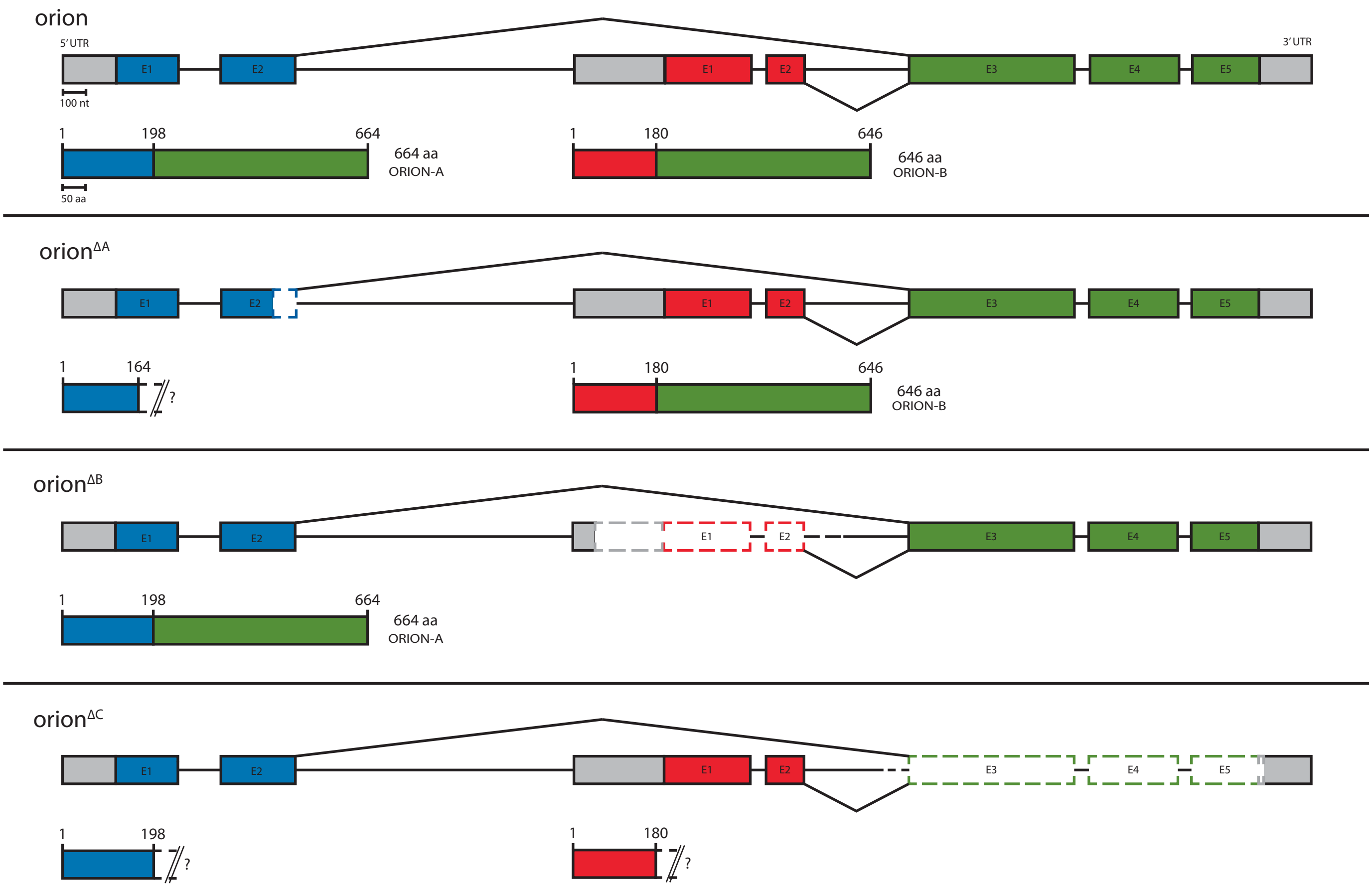

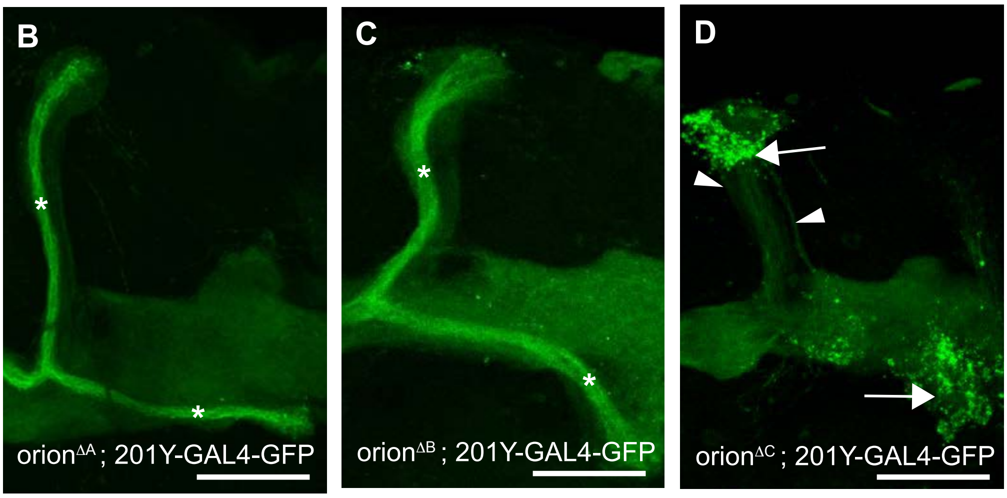

Supplementary Fig. 4. Deletion of the common region of *orion* induces a γ axon pruning phenotype. The *orion* gene (CG2206) lies within the large intron of *Ubr3* and is referenced as *l(1)G0193* although the lethality is clearly not due to the lack of *orion* function (see text) but likely to some splicing defect of the *Ubr3* mRNA induced by the insertion of transposable elements. **a**, Schematic representation of *orion* genomic DNA and its two Orion isoforms in wild-type and in the three different CRISPR induced *orion* deletions (ΔA, ΔB and ΔC) and their corresponding Orion isoforms. **b-d**, Confocal Z-projections of adult MB are revealed by *201Y-GAL4*-driven *UAS-mCD8-GFP* expression (green) in the three *orion* CRISPR mutants: $orion^{\Delta A}$, $orion^{\Delta B}$ *and* $orion^{\Delta C}$ (n = 87, 70 and 98 MBs respectively). Only $orion^{\Delta C}$ displays an unpruned γ axon mutant phenotype characterized by unfragmented γ axons (arrowhead) and uncleared debris (arrow). αβ core axons are labeled by asterisks in **b** and **c** where they are clearly discernible. Scale bars are 40 µm. Genotypes are listed in Supplementary list of fly strains.

Supplementary Fig.5

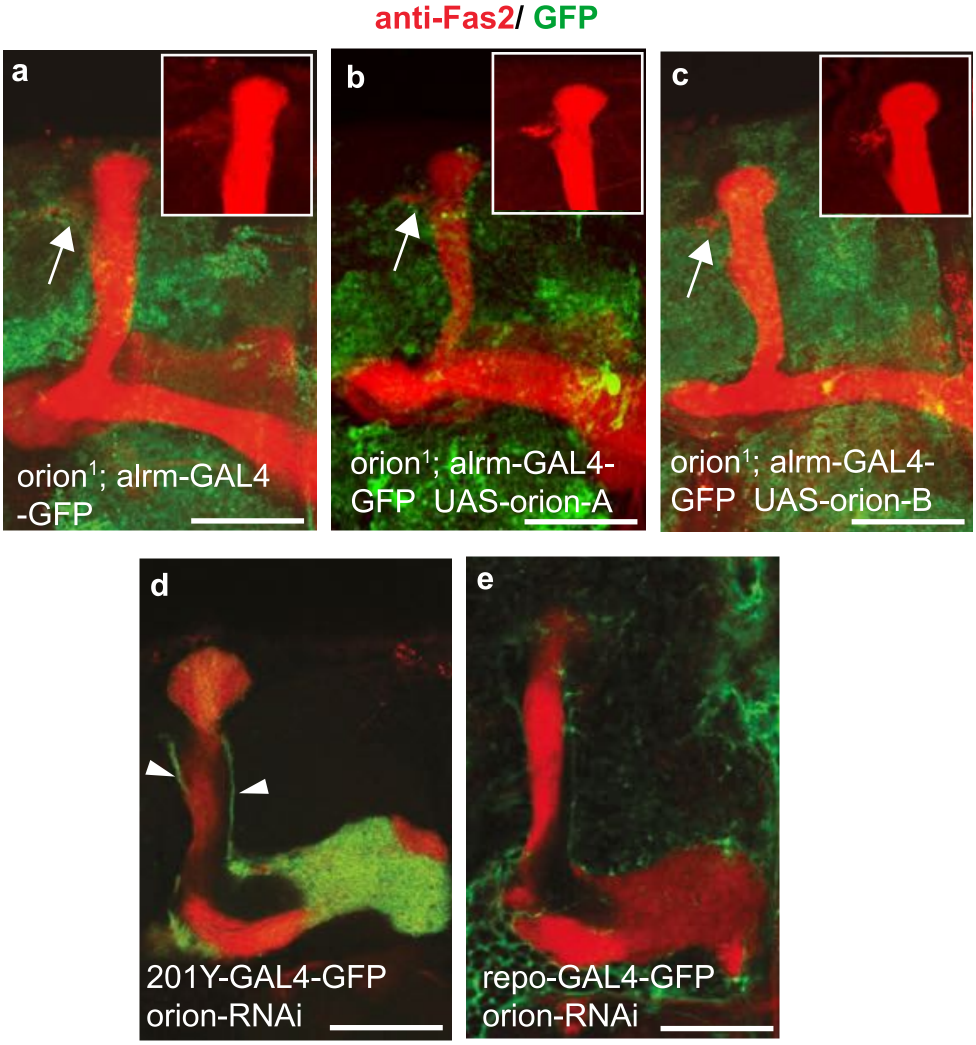

Supplementary Fig. 5. Expression of Orion in glia does not rescue the *orion*^{1} pruning defect and downregulating Orion expression in wild-type glia does not affect pruning. **a-c**, Confocal Z-projections showing merged *alrm-GAL4*-driven *UAS-mCD8-GFP* (green) and anti-Fas2 staining (red) in *orion*^{1} adult MBs. Expression of *orion-A* (**b**) or *orion-B* (**c**) in astrocytes (*alrm-GAL4*) does not rescue the *orion*^{1} mutant phenotype. A rectangle containing anti-Fas2 staining (red) is shown in **a-c**. Arrows point to unpruned γ axons and debris labelled by anti-Fas2 (**a**-**c**). **d**, Confocal plane showing *201Y-GAL4*-driven *UAS-mCD8-GFP* (green) and anti-Fas2 staining (red) in adult MBs. Expression of an *UAS-orion-RNAi* in MB neurons results in an unpruned γ axon phenotype (arrowheads). **e**, Confocal plane showing *repo-GAL4*-driven *UAS-mCD8-GFP* (green) and anti-Fas2 staining (red) in adult MBs. Expression of *orion*-RNAi in glia does not result in unpruned γ axon phenotypes. Scale bars are 40 µm and number of MBs is ≥ 20 (a, n = 20; b, n = 20; c, n = 30; d, n = 20; e, n = 36 MBs). Genotypes are listed in Supplementary list of fly strains.

Supplementary Fig.6

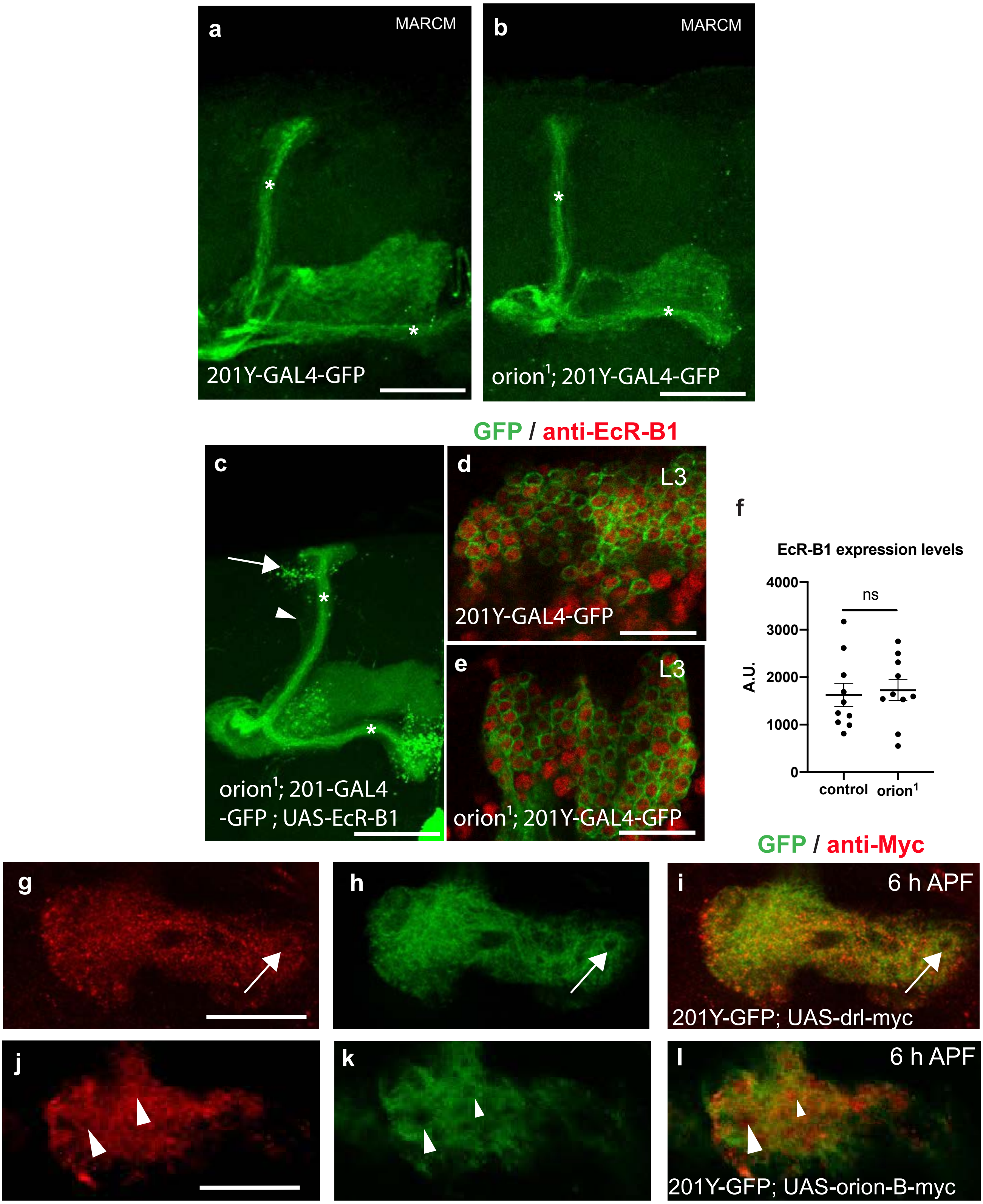

Supplementary Fig. 6. Orion is a secreted protein with a non-cell-autonomous function in γ axons. **a**-**l**, The expression of *201Y-GAL4* driven *UAS-GFP* (green) reveals γ neurons. In adult (**a**-**c**), L3 (**d**, **e**) and 6 h APF (**g**-**l**). **a**, **b**, MARCM neuroblast clones displaying wild-type γ axon pruning are shown. A wild-type control (**a**) and an *orion*^{1} (**b**) (n = 20 wild-type and 30 *orion*^{1} neuroblast clones). **c**, *EcR-B1* expression in *orion*^{1} γ neurons does not rescue the *orion*^{1} phenotype. Note the presence of γ remnant debris (arrow) and unpruned axons (arrowhead) (n = 40 MBs). **d**, **e**, Mushroom body cell body region showing ECR-B1 expression (red staining) in wild-type (**d**) and *orion*^{1} (**e**). **f**, Quantitation of EcR-B1 signal in γ neuron cell bodies in arbitrary units (A.U.) reveals no significant differences between control and *orion*^{1} (results are means ± S.E.M. n = 10 MBs for control and for *orion*^{1}; p = 0.68 (Two-sided Mann-Whitney *U* test)). These interaction analyses support *orion* being genetically downstream of *EcR-B1*. **g**-**l**, Expression of the transmembrane receptor *drl-myc* (**g**-**i**, n = 10) and *orion-B-myc* (**j**-**l**, n = 10) in MBs. Red represents anti-myc staining. Drl-myc staining is absent in hole-like structures (arrows in **g**-**i**). However extracellular Orion-myc is present in these structures (arrowheads in **j**-**l**). Images are confocal Z-projections, except for **g**-**l** which are confocal planes. Scale bars are 40 μm in **a**-**c** and **g**-**l** and 20 μm in **d** and **e**. Genotypes are listed in Supplementary list of fly strains.

Supplementary Fig.7

a

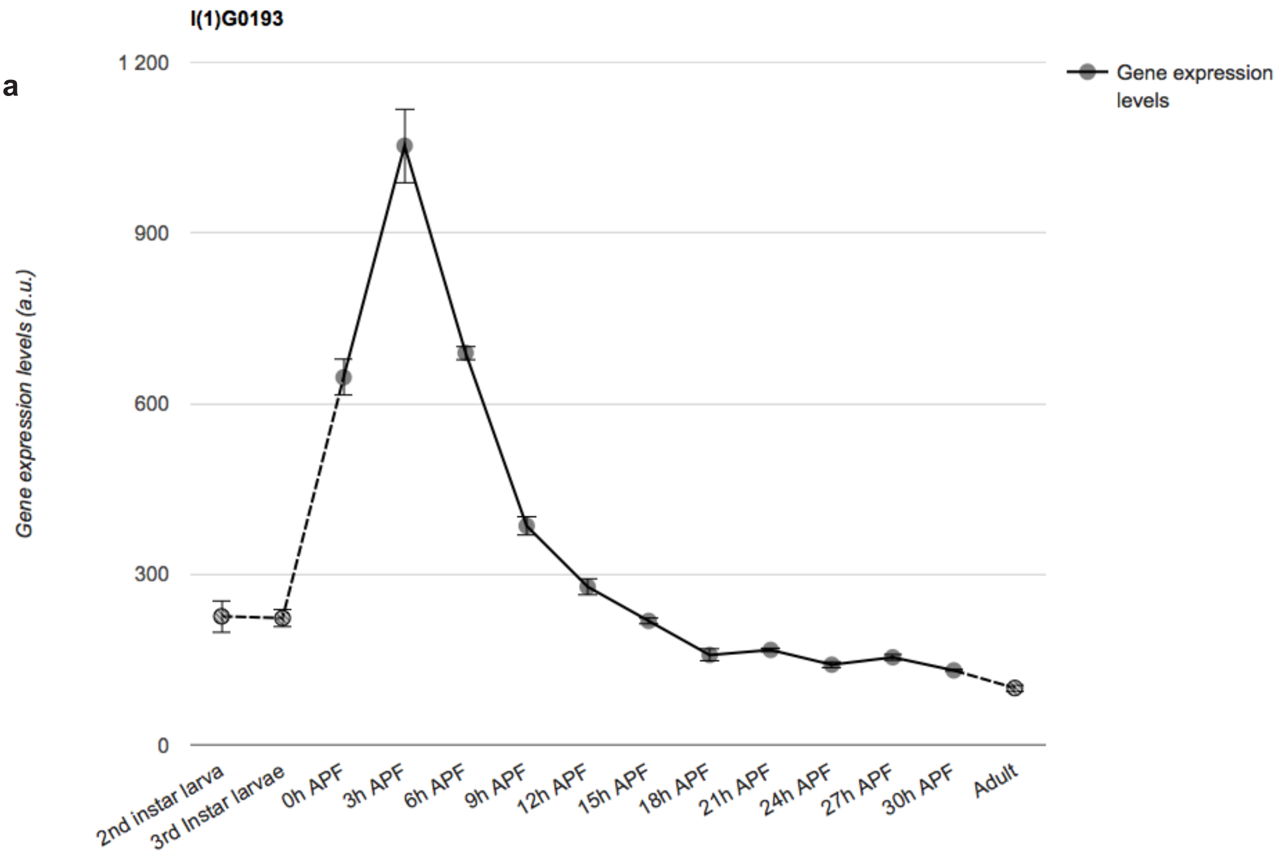

b

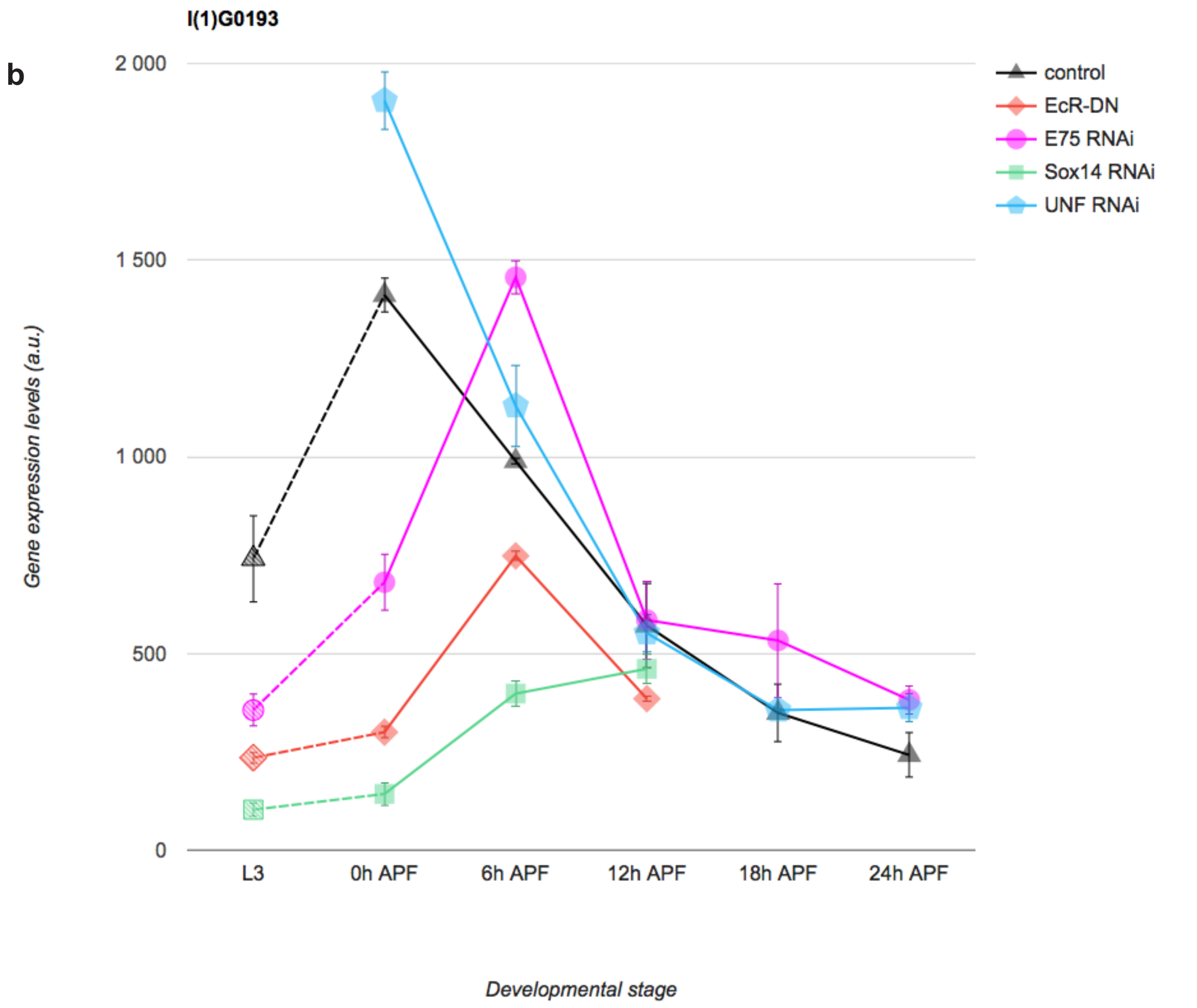

(From Oren Schuldiner's laboratory public web site : https://www.weizmann.ac.il/mcb/Schuldiner/resources)

Supplementary Fig. 7. ***orion*** **mRNA is expressed at the time of pruning in γ neurons and its expression is EcR-B1 regulated.** **a**, **b**, Data showing *orion* mRNA γ neuron-expression levels in arbitrary units (a.u.) during development in wild-type (**a**) and in different mutant backgrounds (**b**), downloaded from Oren Schuldiner's laboratory's public web site (http://www.weizmann.ac.il/mcb/Schuldiner/resources)[24]. Direct web site links are provided as a Source Data file. Note that the peak of expression of *orion* is at 3 h APF which is the timepoint at which pruning initiates (**a**). We also note that just before and during the pruning process (0-6 h APF) *orion mRNA* expression (black line in **b**) is regulated by *EcR-B1* and *Sox14*.

Supplementary Fig.8

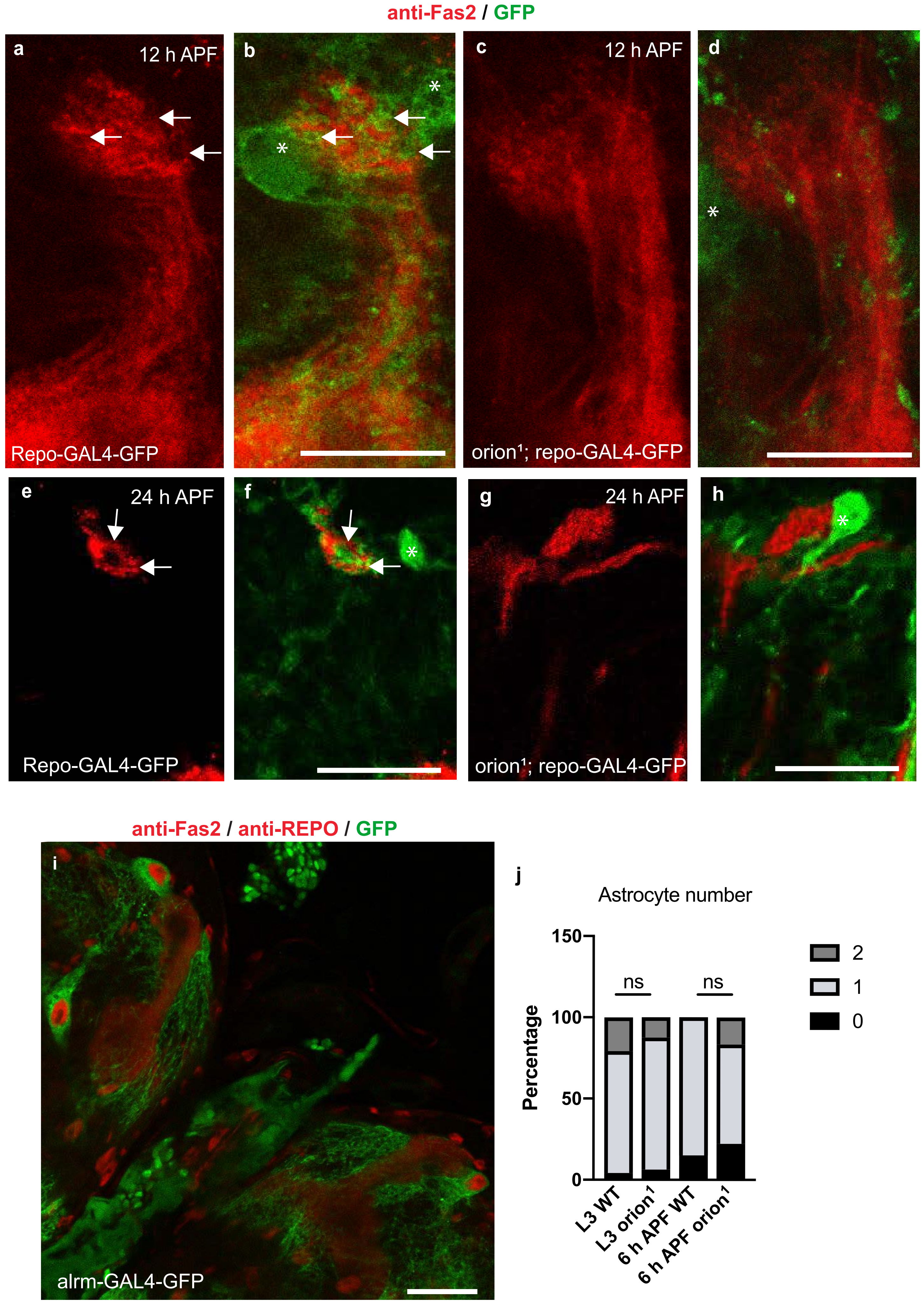

Supplementary Fig. 8. Orion is required for the infiltration of astrocytes into the MB γ bundle and engulfment of degenerated larval axons during development and its mutation does not alter the number of astrocytes surrounding the γ axons.
a-**h**, Confocal Z-projections of 12 h and 24 h APF brains expressing *repo-GAL4*-driven *UAS-mCD8-GFP* (green) in controls (**a**, **b** for 12 h APF and **e, f** for 24 h APF) and *orion*^{1} (**c, d** for 12 h APF and **g, h** for 24 h APF) focused on the MB dorsal lobe (n = 10 control MBs and n = 10 *orion*^{1} MBs). Anti-Fas2 staining (red) reveals spherical hole-like structures occupied by glial processes infiltrating into the γ bundle (green, arrows) in wild-type (**a**, **b** and **e, f**) but not in *orion*^{1} individuals (**c, d** and **g, h**). Note the significant infiltration of the γ bundle by two astrocytes in **b** (asterisk) and the absence of axon bundle infiltration by a closely apposed astrocyte in **d** (asterisk). Note also the lack of remnant axon engulfment by the astrocyte in h (asterisk) compared to f (arrows). Nevertheless, the global aspect of the γ bundle where the fragmentation is taking place looks similar in wild-type and mutant at 12 h APF. This suggests that, in *orion* mutant, fragmenting γ axons are not actively being engulfed by astrocytes. Scale bars are 20 µm. **i**, Confocal Z-projection showing *UAS-mCD8-GFP* expression (green) in astrocytes driven by *alrm-GAL4* at L3. Red shows both glial cell nuclei labelled by an anti-Repo antibody and MBs labelled by anti-Fas2. Scale bars are 40 µm. **j**, Percentage of astrocytes surrounding the γ vertical lobe at L3 and at 6 h APF in wild-type and *orion*^{1} (for L3, n = 24 wild-type MBs and n = 16 *orion*^{1} MBs; for 6 h APF, n = 20 wild-type MBs and n =18 *orion*^{1} MBs). No statistically-significant differences were observed between the two groups (Two-sided Fisher's exact test p = 0.84 for L3 and p = 0.12 for 6 h APF). Scale bar is 30 µm. Source data are provided as a Source Data file. Genotypes are listed in Supplementary list of fly strains.

Supplementary Fig.9

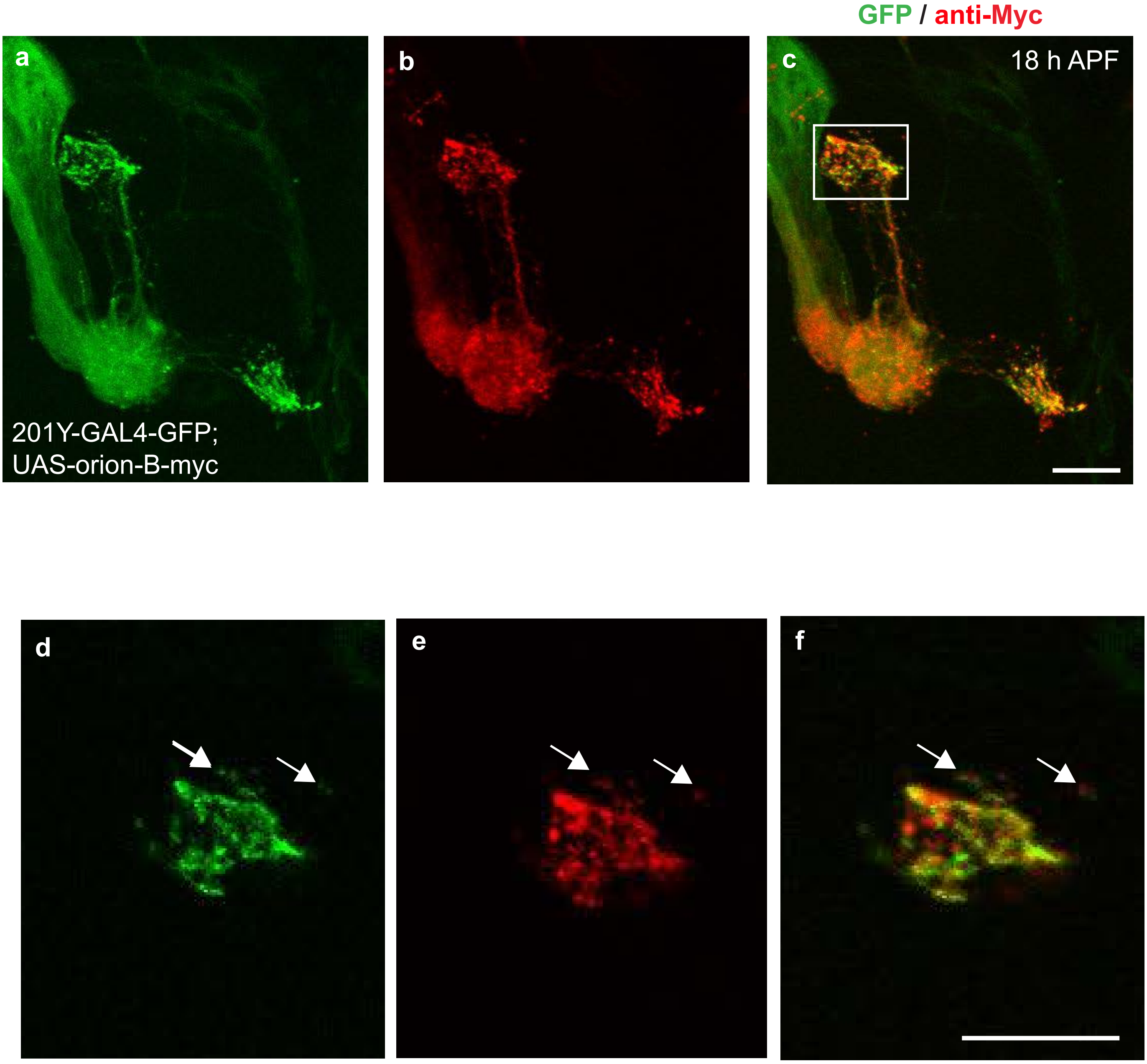

Supplementary Fig. 9. Orion is associated with membranes. a-c, Expression of *UAS-mCD8-GFP* (green) and *UAS-orion-B-myc* (red) under the control of *201Y-GAL4* is shown in 18 h APF γ neurons (n = 6 MBs). **d-f**, (confocal planes) are higher magnifications of the **a-c** (confocal Z-projections) regions enclosed by rectangles. Some debris staining for both GFP and Orion-Myc is labelled by arrows. Scale bars are 20 µm. Genotypes are listed in Supplementary list of fly strains.

Supplementary Fig.10

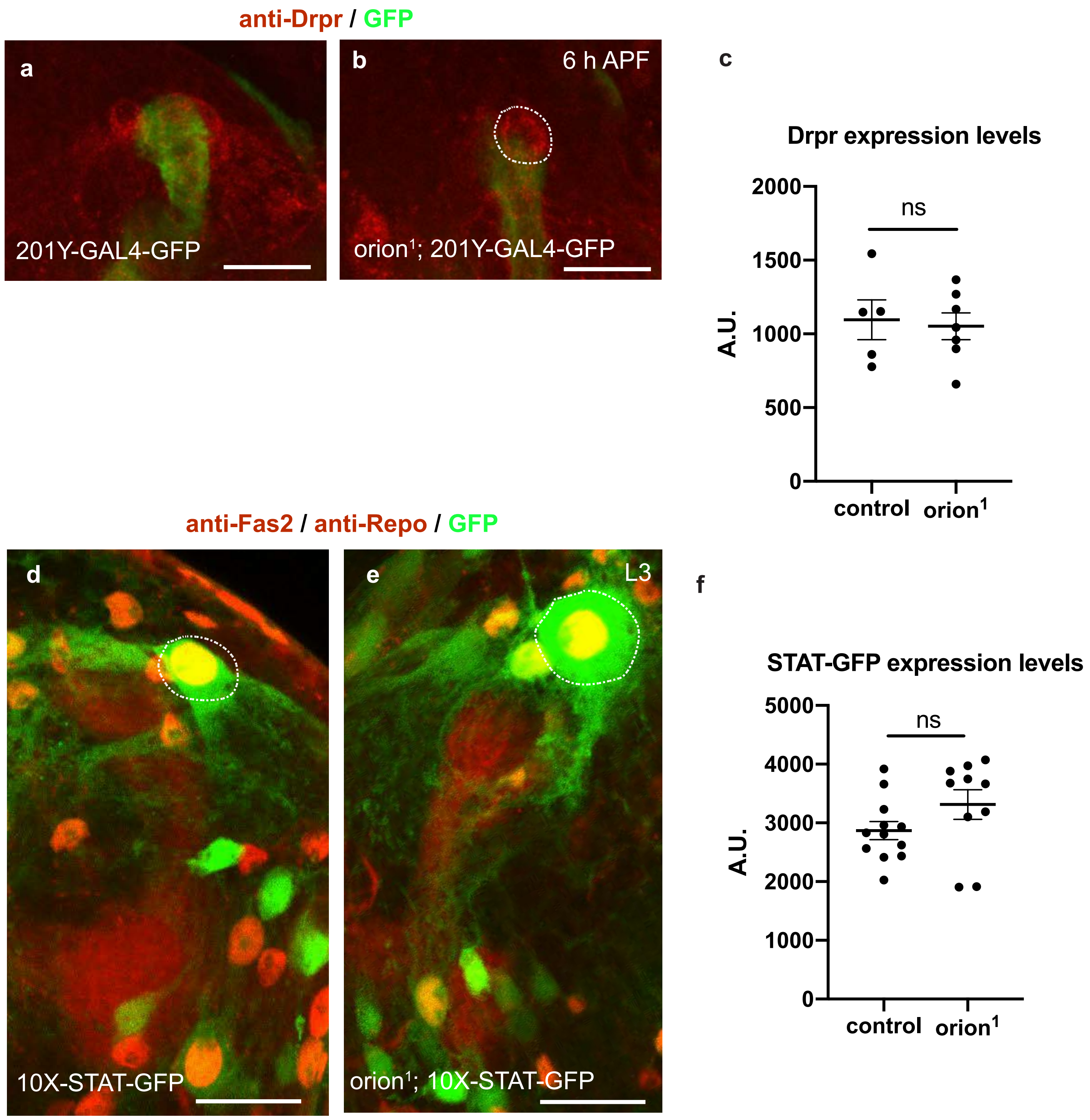

Supplementary Fig. 10. *orion*^{1} mutants display wild-type levels of Drpr protein expression and wild-type activity of the *drpr* transcriptional regulator STAT92E. Since *drpr* expression is regulated by known axon degeneration cues[31,34] and the *drpr*$^{\Delta 5}$ and *orion*^{1} alleles display mutant phenotypes that share some features (Supplementary Fig.1), we analyzed Drpr expression in wild-type and *orion*^{1}. **a**-**c**, Expression of Drpr (red) in wild-type control (**a**) and *orion*^{1} (**b**) in 6 h APF brains and the corresponding quantitation in arbitrary units (A.U.) in astrocytes (**c**). Green corresponds to the expression of *201Y-GAL4* driven *UAS-mCD8-GFP* in γ axons. The astrocyte cytoplasm, in which quantitation are performed, is circled by a white dotted line in a and b. **c**, Quantitation of Drpr expression in arbitrary units (A.U.) reveals no significant differences between control and *orion*^{1}. Results are means ± S.E.M. n = 5 MBs for control and 7 MBs for *orion*^{1}; p = 1 (Two-sided Mann-Whitney *U* test). Source data are provided as a Source Data file. In addition, since *drpr* expression is regulated by the transcription factor STAT92E[47], we analyzed the expression of an *STAT92E-GFP* reporter in wild-type and *orion*^{1}. **d**-**f**, Expression of 10X-STAT92E-GFP (green) in wild-type control (**d**) and *orion*^{1} (**e**) larval brains and the corresponding quantitation in arbitrary units (A.U.) **(f).** Red is both, Fas2 labelling γ axon bundles and Repo antibody labelling the glial cell nuclei. Pictures are confocal Z-projections. The astrocyte cytoplasm, in which quantitation are performed, is circled by a white dotted line in **d** and **e**. **f**, Quantitation of STAT-GFP expression in arbitrary units (A.U.) reveals no significant differences between control and *orion*^{1}. Results are means ± S.E.M. n = 12 MBs for control and 10 MBs for *orion*^{1}; p = 0.09 (Two-sided Mann-Whitney *U* test). Source data are provided as a Source Data file. Scale bars are 30 μm in **a**, **b** and 20 μm in **d**, **e**. Genotypes are listed in Supplementary list of fly strains.

Supplementary list of fly strains:

Fig. 1: a, c-e, *y* w^{67c23} / Y or *y* w^{67c23} / *y* w^{67c23}; *UAS-mCD8GFP 201Y-GAL4/+*. **b**, **f-i**, *y* w^{67c23} sn^3 *orion* ^{1} *FRT19A* / Y or *y* w^{67c23} sn^3 *orion* ^{1} *FRT19A* / *y* w^{67c23} sn^3 *orion* ^{1} *FRT19A; UAS-mCD8GFP 201Y-GAL4* / +. **j**, *y* w^{67c23} sn^3 $orion^1$ *FRT19A* / Y; *UAS-mCD8GFP 201Y-GAL4/+; UAS-orion-A-myc* / +. **k**, *y* w^{67c23} sn^3 $orion^1$ *FRT19A* / Y; *UAS-mCD8GFP 201Y-GAL4* / +; *UAS-orion-B-myc* / +. **l**, *y* w^{67c23} / *Y* or *y* w^{67c23} / *w* ^{1118}; *UAS-mCD8GFP 201Y-GAL4 / UAS-orion-RNAi*.

Fig. 3: a, **b**, **d**, **e**, **g**, **h**, **j, k,** *y* w^{67c23} / Y or *y* w^{67c23} /*y w** ; *UAS-mCD8GFP 201Y-GAL4* / + ; *UAS-orion-B-myc* / +. **c, f, i,** *y* w^{67c23} / Y or *y* w^{67c23} /*y w** ; *UAS-mCD8GFP 201Y-GAL4* / + ; *UAS-orion-B-ΔSP-myc* / +.

Fig. 4: a, **b**, *y* w^{67c23} sn^3 *FRT19A* / Y ; *repo-GAL4 UAS-mCD8GFP* / +. **c**, *y* w^{67c23} sn^3 $orion^1$ *FRT19A* / Y ; *repo-GAL4 UAS-mCD8GFP* / +. **d-g**, *y* w^{67c23} / Y *or y* w^{67c23} / *y* w^* ; *UAS-mCD8GFP 201Y-GAL4* / + ; *UAS-orion-B-myc* / +. **h**, *y* w^{67c23} / Y *or y* w^{67c23} / *y* w^*; *UAS-mCD8GFP 201Y-GAL4* / + ; *UAS-orion-B-ΔSP-myc* / +.

Supplementary Fig. 1: a, e, h-k, *y* w^{67c23} / Y or *y* w^{67c23} / *y* w^{67c23}; *UAS-mCD8GFP 201Y-GAL4* / +. **b, l-o,** *y* w^{67c23} sn^3 $orion^1$ *FRT19A* / Y *or y* w^{67c23} sn^3 $orion^1$ *FRT19A* / *y* w^{67c23} sn^3 $orion^1$; *UAS-mCD8GFP 201Y-GAL4* / +. **c**, *y* w^{67c23} / Y or *y* w^{67c23} / *y* w^{67c23} ; *UAS-mCD8GFP 201Y-GAL4* / + ; $drpr^{\Delta5}$ / $drpr^{\Delta5}$. **d, g**, *y* w^{67c23} / Y or *y* w^{67c23}/ *y* w^{67c23}; $Hr39^{C13}$, *UAS-mCD8GFP 201Y-GAL4* / +. **f**, *y* w^* $orion^{\Delta C}$ / Y ; *UAS-mCD8GFP 201Y-GAL4* / +. **p**, *y* w^{67c23} sn^3 $orion^1$ *FRT19A* / Y ; *UAS-mCD8GFP 201Y-GAL4* / + ; *UAS-orion-B-myc* / +.

Supplementary Fig. 2: a, e and j. WT: *y* w^{67c23} / Y; *UAS-mCD8GFP 201Y-GAL4* / +. **Hr39**: *y* w^{67c23} / Y; $Hr39^{C13}$, *UAS-mCD8GFP 201Y-GAL4* / +. **orionΔC**: *y* w^* $orion^{\Delta C}$ / Y ; *UAS-mCD8GFP 201Y-GAL4* / +. **orion1**: *y* w^{67c23} sn^3 $orion^1$ *FRT19A* / Y ; *UAS-mCD8GFP 201Y-GAL4* / +. **orionRNAi**: *y* w^{67c23} / *Y; UAS-mCD8GFP 201Y-GAL4 / UAS-orion-RNAi*. **drprΔ5**: *y* w^{67c23} / Y; *UAS-mCD8GFP 201Y-GAL4* / + ; $drpr^{\Delta5}$ / $drpr^{\Delta5}$. **b, f,** *y* w^{67c23} / Y; *UAS-mCD8GFP 201Y-GAL4* / +. **c,** *y* w^{67c23} sn^3 $orion^1$ *FRT19A* / Y ; *UAS-mCD8GFP 201Y-GAL4* / +. **d:** *y* w^{67c23} / Y; $Hr39^{C13}$, *UAS-mCD8GFP 201Y-GAL4* / +. **g-i, k-m,** *UAS-mCD8GFP 201Y-GAL4* / + ; $drpr^{\Delta5}$ / $drpr^{\Delta5}$. **n**, *y* w^* $orion^{\Delta C}$ / Y ; *UAS-mCD8GFP 201Y-GAL4* / +.

Supplementary Fig. 3: **a**, *y* w^{67c23} sn^{3} $orion^{1}$ *FRT19A* / Y; *UAS-mCD8GFP 201Y-GAL4 / +; UAS-orion-B-myc / +*. **b**, *y* w^{67c23} sn^{3} $orion^{1}$ *FRT19A* / Y ; *UAS-mCD8GFP 201Y-GAL4 / +; UAS-orion-B- Mut AX3C-myc / +*. **c**, *y* w^{67c23} sn^{3} $orion^{1}$ *FRT19A* / Y ;*UAS-mCD8GFP 201Y-GAL4 / + ; UAS-orion-B-Mut CX4C-myc- /* +. **d,** *y* w^{67c23} sn^{3} $orion^{1}$ *FRT19A* / Y; *UAS-mCD8GFP 201Y-GAL4 / + ; UAS-orion-B-ΔSP-myc / +*. **e**, *y* w^{67c23} sn^{3} $orion^{1}$ *FRT19A* / Y; *UAS-mCD8GFP 201Y-GAL4 / +; UAS-orion-B-Mut GAG1-myc / +*. **f**, *y* w^{67c23} sn^{3} $orion^{1}$ *FRT19A* / Y; *UAS-mCD8GFP 201Y-GAL4 / +; UAS-orion-B-Mut GAG2-myc* / +. **g**, *y* w^{67c23} sn^{3} $orion^{1}$ *FRT19A* / Y; *UAS-mCD8GFP 201Y-GAL4 / +; UAS-orion-B-Mut GAG3-myc / +*. **h**, **control**: *y* w^{67c23} / Y; *UAS-mCD8GFP 201Y-GAL4 / +*. **orion1**: *y* w^{67c23} sn^{3} $orion^{1}$ *FRT19A* / Y; *UAS-mCD8GFP 201Y-GAL4 / +*. **orion1 + orion-B WT**: see above (**a**). **orion1 + ΔSP**: see above (**d**). **orion1 + AX3C**: see above (**b**). **orion1 + CX4C**: see above (**c**). **orion1 + GAG1**: see above (**e**). **orion1 + GAG2**: see above (**f**). **orion1 + GAG3**: see above (**g**). **orion-RNAi:** *y* w^{67c23} / *Y; UAS-mCD8GFP 201Y-GAL4 / UAS-orion-RNAi*. **orion-RNAi + EcR-B1**: *y* w^{67c23} / *Y; UAS-mCD8GFP 201Y-GAL4 / UAS-orion-RNAi ; UAS-EcR-B1/+*. **orion-RNAi + control** : *y* w^{67c23} / *Y; UAS-mCD8GFP 201Y-GAL4 / UAS-orion-RNAi ; UAS-FRT-*y^{+}*-FRT/+*. **i**, 1 : *y* w^{67c23} sn^{3} $orion^{1}$ *FRT19A* / Y; *UAS-mCD8GFP 201Y-GAL4 / +; UAS-orion-A-myc / +*. 2 : *y* w^{67c23} sn^{3} $orion^{1}$ *FRT19A* / Y; *UAS-mCD8GFP 201Y-GAL4 / +; UAS-orion-B-myc / +*. 3 : *y* w^{67c23} sn^{3} $orion^{1}$ *FRT19A* / Y ; *UAS-mCD8GFP 201Y-GAL4 / + ; UAS-orion-B -ΔSP-myc / +*. 4 : *y* w^{67c23} sn^{3} $orion^{1}$ *FRT19A* / Y ; *UAS-mCD8GFP 201Y-GAL4 / +; UAS-orion-B-Mut AX3C-myc / +*. 5 : *y* w^{67c23} sn^{3} $orion^{1}$ *FRT19A* / Y ;*UAS-mCD8GFP 201Y-GAL4 / + ; UAS-orion-B-Mut CX4C-myc/+*. 6 : *y* w^{67c23} sn^{3} $orion^{1}$ *FRT19A* /Y; *UAS-mCD8GFP 201Y-GAL4 / +; UAS-orion-B-Mut GAG1-myc / +*. 7 : *y* w^{67c23} sn^{3} $orion^{1}$ *FRT19A* /Y; *UAS-mCD8GFP 201Y-GAL4 / +; UAS-orion-B -Mut GAG2-myc / +*. 8 : *y* w^{67c23} sn^{3} $orion^{1}$ *FRT19A* /Y; *UAS-mCD8GFP 201Y-GAL4 / +; UAS-orion-B -Mut GAG3-myc / +*.

Supplementary Fig. 4: **b**, *y* w^{*} $orion^{\Delta A}$ / Y ; *UAS-mCD8GFP 201Y-GAL4 / +*. **c**, *y* w^{*} $orion^{\Delta B}$/ Y ; *UAS-mCD8GFP 201Y-GAL4 / +*. **d**, *y* w^{*} $orion^{\Delta C}$ / Y ; *UAS-mCD8GFP 201Y-GAL4 / +*.

Supplementary Fig. 5: **a**, y w^{67c23} sn^{3} $orion^{1}$ *FRT19A* / Y; *CyO, P(Dfd-GMR-nvYFP)2 /+* or *Sp / + ; alrm-GAL4 UAS-mCD8GFP / +*. **b**, *y* w^{67c23} sn^{3} $orion^{1}$ *FRT19A* / Y ; *CyO, P(Dfd-GMR-nvYFP)2 /+* or *Sp / + ; alrm-GAL4 UAS-mCD8GFP / UAS-orion-A-myc*. **c**, *y* w^{67c23} sn^{3} $orion^{1}$ *FRT19A* / Y ; *CyO, P(Dfd-GMR-nvYFP)2 /+* or *Sp / + ; alrm-GAL4 UAS-mCD8GFP / UAS-orion-B-myc*. **d**, *y* w^{67c23} / Y or *y* w^{67c23} / w^{*} ; *UAS-mCD8GFP 201Y-GAL4 / UAS-orion-RNAi*. **e**, w^{*}/ Y or w^{*} / w^{*} ; *UAS-orion-RNAi/+; repo-GAL4 UAS-mCD8GFP/+*.

Supplementary Fig. 6: a, *w* tub-P-GAL80 hs-FLP122 FRT19A / y* w^{67c23} sn^3 *FRT19A ; UAS-mCD8GFP 201Y-GAL4 / +.* **b**, *w* tub-P-GAL80 hs-FLP122 FRT19A / y* w^{67c23} sn^3 $orion^1$ *FRT19A ; UAS-mCD8GFP 201Y-GAL4 / +.* **c**, *y* w^{67c23} sn^3 $orion^1$ *FRT19A* / Y *; UAS-mCD8GFP 201Y-GAL4 / + ; UAS-EcR-B1 / +.* **d**, *y* w^{67c23} sn^3 *FRT19A* / Y ; *UAS-mCD8GFP 201Y-GAL4 / +.* **e**, *y* w^{67c23} sn^3 $orion^1$ *FRT19A* / Y *; UAS-mCD8GFP 201Y-GAL4 / +.* **g-i**, *y* w^{67c23} / Y or *y* w^{67c23} / *y* w^{67c23} *; UAS-mCD8GFP 201Y-GAL4 / + ; 2x UAS-drl-myc / +.* **j-l**, *y* w^{67c23} / Y *;* or *y* w^{67c23} / *y* w^{*} *UAS-mCD8GFP 201Y-GAL4 / + ; UAS-orion-B-myc / +.*

Supplementary Fig. 8: a, **b, e, f**, *y* w^{67c23} sn^3 *FRT19A* / Y *; repo-GAL4 UAS-mCD8GFP / +.* **c, d, g, h** *y* w^{67c23} sn^3 $orion^1$ *FRT19A* / Y *; repo-GAL4 UAS-mCD8GFP / +.* **i**, *w* / Y or w* / w* ; CyO, P(Dfd-GMR-nvYFP)2 / Sp ; alrm-GAL4 UAS-mCD8GFP / alrm-GAL4 UAS-mCD8GFP.* **j**, WT : *y* w^{67c23} */ Y ; CyO, P(Dfd-GMR-nvYFP)2 / +* or *Sp / + ; alrm-GAL4 UAS-mCD8GFP /+.* $orion^1$: *y* w^{67c23} sn^3 $orion^1$ *FRT19A / Y ; CyO, P(Dfd-GMR-nvYFP)2 / +* or *Sp / + ; alrm-GAL4 UAS-mCD8GFP / +.*

Supplementary Fig. 9: a-f, *y* w^{67c23} / Y or *y* w^{67c23} */ y* w^{*} *; UAS-mCD8GFP 201Y-GAL4 / + ; UAS-orion-B-myc / +.*

Supplementary Fig. 10: a, *y* w^{67c23} / Y or *y* w^{67c23} */ y* w^{67c23} *; UAS-mCD8GFP 201Y-GAL4 / CyO.* **b**, *y* w^{67c23} sn^3 $orion^1$ *FRT19A* / Y *or y* w^{67c23} sn^3 $orion^1$ *FRT19A /y* w^{67c23} sn^3 $orion^1$ *FRT19A ; UAS-mCD8GFP 201Y-GAL4 / CyO.* **d**, *y* w^{67c23} sn^3 *FRT19A* / Y ; *10X-STAT92E-GFP / +.* **e**, *y* w^{67c23} sn^3 $orion^1$ *FRT19A / Y ; 10X-STAT92E-GFP / +.*

Supplementary Table I

**Oligonucleotide name**	**Oligonucleotide sequence 5' to 3'**
CRISPR-1 orion A fwd	TATATAGGAAAGATATCCGGGTGAACTTCATTTGCGTTTTGATTTTCAGGTTTTAGAGCTAGAAATAGCAAG
CRISPR-1 orion A rev	ATTTTAACTTGCTATTTCTAGCTCTAAAACGCTGTTGGAGTAGATTGGTGGACGTTAAATTGAAAATAGGTC
CRISPR-1 orion B fwd	TATATAGGAAAGATATCCGGGTGAACTTCGTGAAATCTCAGCTGTATCGGTTTTAGAGCTAGAAATAGCAAG
CRISPR-1 orion B rev	ATTTTAACTTGCTATTTCTAGCTCTAAAACGCTAGATTTAAAACGGCAAGGACGTTAAATTGAAAATAGGTC
CRISPR-1 orion common region C fwd	TATATAGGAAAGATATCCGGGTGAACTTCACCTGGTAAAGAATGCCAGAGTTTTAGAGCTAGAAATAGCAAG
CRISPR-1 orion common region C rev	ATTTTAACTTGCTATTTCTAGCTCTAAAACCTTCGCGTCCAGGTGAGTCTGACGTTAAATTGAAAATAGGTC
orion-A fwd	CACCAAAACATGAGATTTATAAATTGGGTACTTCCCCT
orion-B fwd	CACCAAAACATGGCGCCGCCTTTCGGATTATTA
orion-AB rev	GAATCTATTCTTTGGCACCTGAACGT
orion-B ΔSP fwd	CACCAAAACATGGGAAATACAAAATCGGCGTCGTCCG
