# Supplementary material for: Axonal chemokine-like Orion induces astrocyte infiltration and engulfment during mushroom body neuronal remodeling
Source: Nat Commun. 2021 Mar 23;12:1849. doi: 10.1038/s41467-021-22054-x (PMC7988174; doi:10.1038/s41467-021-22054-x)
Supplement: Supplementary file 3 — Reporting Summary [file 41467_2021_22054_MOESM3_ESM.pdf]

nature research

Corresponding author(s): Jean-Maurice DURA

Last updated by author(s): Feb 22, 2021

# Reporting Summary

Nature Research wishes to improve the reproducibility of the work that we publish. This form provides structure for consistency and transparency in reporting. For further information on Nature Research policies, see Authors & Referees and the Editorial Policy Checklist .

## Statistics

For all statistical analyses, confirm that the following items are present in the figure legend, table legend, main text, or Methods section.

| n/a | Confirmed | |
|---|---|---|
| ☐ | ☒ | The exact sample size (*n*) for each experimental group/condition, given as a discrete number and unit of measurement |
| ☐ | ☒ | A statement on whether measurements were taken from distinct samples or whether the same sample was measured repeatedly |
| ☐ | ☒ | The statistical test(s) used AND whether they are one- or two-sided<br>*Only common tests should be described solely by name; describe more complex techniques in the Methods section.* |
| ☒ | ☐ | A description of all covariates tested |
| ☒ | ☐ | A description of any assumptions or corrections, such as tests of normality and adjustment for multiple comparisons |
| ☐ | ☒ | A full description of the statistical parameters including central tendency (e.g. means) or other basic estimates (e.g. regression coefficient) AND variation (e.g. standard deviation) or associated estimates of uncertainty (e.g. confidence intervals) |
| ☐ | ☒ | For null hypothesis testing, the test statistic (e.g. *F*, *t*, *r*) with confidence intervals, effect sizes, degrees of freedom and *P* value noted<br>*Give P values as exact values whenever suitable.* |
| ☒ | ☐ | For Bayesian analysis, information on the choice of priors and Markov chain Monte Carlo settings |
| ☒ | ☐ | For hierarchical and complex designs, identification of the appropriate level for tests and full reporting of outcomes |
| ☒ | ☐ | Estimates of effect sizes (e.g. Cohen's *d*, Pearson's *r*), indicating how they were calculated |

*Our web collection on statistics for biologists contains articles on many of the points above.*

## Software and code

Policy information about availability of computer code

| | |
|---|---|
| Data collection | Microscopy: Zeiss LSM 780 laser scanning confocal<br>Zen 2011 (black edition) software |
| Data analysis | Microscopy: Image J v1.51, Fiji and Imaris v9.1.2 (Bitplane)<br>Graphs and statistics: GraphPad Prism version 8.1.1<br>PrediSi version 2003<br>TMHMM version2.0e40 |

For manuscripts utilizing custom algorithms or software that are central to the research but not yet described in published literature, software must be made available to editors/reviewers. We strongly encourage code deposition in a community repository (e.g. GitHub). See the Nature Research guidelines for submitting code & software for further information.

## Data

Policy information about availability of data

All manuscripts must include a data availability statement. This statement should provide the following information, where applicable:

- Accession codes, unique identifiers, or web links for publicly available datasets
- A list of figures that have associated raw data
- A description of any restrictions on data availability

The authors declare that the data supporting the findings of this study are available within the paper and supplementary information files. The source data underlying Figs. 4g-j and Supplementary Figs 3i and j, 8j and 10c, f are provided as a Source Data file.

# Field-specific reporting

Please select the one below that is the best fit for your research. If you are not sure, read the appropriate sections before making your selection.

- [x] Life sciences
- [ ] Behavioural & social sciences
- [ ] Ecological, evolutionary & environmental sciences

For a reference copy of the document with all sections, see nature.com/documents/nr-reporting-summary-flat.pdf

# Life sciences study design

All studies must disclose on these points even when the disclosure is negative.

**Sample size**

The sample sizes were determined empirically on the basis of observed effects and are similar to those reported in previous publications.

**Data exclusions**

No Data were excluded

**Replication**

All attempts at replication were successful.

In all experiments at least two replicates were done except for the following Supplementary Figures.

Supplementary Fig. 1p which involved the rescue of the orion dendrite phenotype at 18h APF: All the 8 MBs were similar and the rescue was already observed in replicas in adults (Figure 1K).

Supplementary Fig. 3i-j: This experiment was an additional control to validate the transgene expression already seen by immunofluorescence.

Supplementary Fig. 5b which involved UAS-orion-A: All the 20 MBs were similar and the similar result was already obtained in replicas with UAS-orion-B (Supplementary Fig. 5c).

Supplementary Fig. 6c which involved the absence of rescue of orion phenotype by UAS-EcR-B1: All the 40 MBs were similar. The UAS-EcR-B1 was validated by the rescue of orion RNAi (Supp Fig.3h).

Supplementary Fig. 6d and e : Although the quantitation was done only once, the corresponding experiments were done in replicas and we did not observe differences in staining intensities with the fluorescence microscope.

Supplementary Fig. 9 : All the 6 MBs at 18h APF were similar and the similar result was already observed in replicas at 6h APF (Figure 3j, k).

Supplementary Fig. 10 : Although the quantitation was done only once, the corresponding experiments were done in replicas and we did not observe differences in staining intensities with the fluorescence microscope.

**Randomization**

This is not relevant to our study because we compared samples with different genetic backgrounds.

**Blinding**

This is not relevant to our study because samples were not allocated to groups.

# Reporting for specific materials, systems and methods

We require information from authors about some types of materials, experimental systems and methods used in many studies. Here, indicate whether each material, system or method listed is relevant to your study. If you are not sure if a list item applies to your research, read the appropriate section before selecting a response.

## Materials & experimental systems

| n/a | Involved in the study | |
|---|---|---|
| [ ] | [x] | Antibodies |
| [x] | [ ] | Eukaryotic cell lines |
| [x] | [ ] | Palaeontology |
| [ ] | [x] | Animals and other organisms |
| [x] | [ ] | Human research participants |
| [x] | [ ] | Clinical data |

## Methods

| n/a | Involved in the study | |
|---|---|---|
| [x] | [ ] | ChIP-seq |
| [x] | [ ] | Flow cytometry |
| [x] | [ ] | MRI-based neuroimaging |

# Antibodies

**Antibodies used**

anti-Fas2 (1D4; 1:10; Developmental Studies Hybridoma Bank; University of Iowa, Iowa, IA, USA).

anti-Drpr (8A1; 1:400; Developmental Studies Hybridoma Bank; University of Iowa, Iowa, IA, USA).

anti-Repo (8D1.2; 1:400; Developmental Studies Hybridoma Bank ; University of Iowa, Iowa, IA, USA).

anti-EcR-B1 (4D4.4; 1:5000; Developmental Studies Hybridoma Bank; University of Iowa, Iowa, IA, USA).

anti-Myc (9E10; 1:1000; cat # ab32, lot #GR206680-1  Abcam, Cambridge, MA USA).

anti-Myc (71D10; 1:500; cat # 2278S lot # 5 was purchased from Cell Signaling Technology, Danvers, MA, USA).

Anti-Tubulin (cat # T5168 lot # 038M4813V was purchased from SIGMA-Aldrich, Darmstadt, Germany).

Goat anti-mouse IgG (H+L) Secondary Antibody, Cy3-conjugated (1:300; cat  # 115-166-006; lot  # 122298; Jackson ImmunoResearch Laboratories, Inc. PA, USA

Goat anti-rabbit IgG (H+L) Secondary Antibody, Cy3-conjugated (1:300; cat # 711-165-152; lot # 111674; Jackson ImmunoResearch Laboratories, Inc. PA, USA

Goat anti-mouse IgG (H+L) Secondary Antibody, Alexa Fluor 647-conjugated (1:300; cat # A21236; lot # 54415A; ThermoFisher Scientific; Waltham MA.

Donkey anti-rabbit IgG (H+L) Secondary Antibody, Cy5-conjugated (1:300; cat # 711-175-152; lot # 110231; Jackson ImmunoResearch Laboratories, Inc. PA, USA.

Peroxidase-conjugated AffiniPure Goat Anti-Mouse Secondary Antibody cat # 115-035-146 lot # 111325; Jackson ImmunoResearch Laboratories, Inc. PA, USA.

Validation

Antibodies were selected according to the antibody validation profiles reported by the distributing companies and in publications:
anti-Fas2 (species Mouse): Cited by 64 publications on manufacturer's website.

anti-Drpr (species Mouse): Cited by 4 publications on manufacturer's website. In one of these citations: Astrocytes play a key role in Drosophila mushroom body axon pruning. Schuldiner O, PloS one 9.1 (2014): e86178; authors use this antibody to label mushroom bodies surrounding astrocytes during development.

anti-Repo (species Mouse): Cited in Nature Communications on 3 July 2015 by Erdmann, I., Marter, K., et al.. on manufacturer's website.

anti-EcR-B1 (species Mouse). Cited by 3 publications on manufacturer's website. In addition, we previously validated this antibody and quantify EcR-B1 expression in mushroom body Kenyon cells: ftz-f1 and Hr39 opposing roles on EcR expression during Drosophila mushroom body neuron remodeling. Boulanger et al., Nat Neurosci. 2011 Jan;14(1):37-44.

anti-Myc (species Mouse): Used in this study for both immunohistochemistry and western blots. This antibody was reported in 283 citations on Abcam manufacturer's website. From these, in 67 citations this antibody was successfully used in regular western blots experiments.

anti-Myc (species Rabbit): Cited by 308 publications on Cell Signaling manufacturer's website.

Anti-Tubulin (species Mouse): Cited by 2567 publications on Cell Signaling manufacturer's website.

# Animals and other organisms

Policy information about studies involving animals; ARRIVE guidelines recommended for reporting animal research

Laboratory animals

We used Drosophila melanogaster laboratory stocks. Third instar larvae, 0 to 24 hours after puparium formation pupae and one to 10 days old adults (both females and males) were analyzed.

Wild animals

No wild animals were used in this study.

Field-collected samples

No field collected samples were used in this study.

Ethics oversight

This study did not require an ethical approval since laboratory animals were insects.

Note that full information on the approval of the study protocol must also be provided in the manuscript.
